# An ethnobotanical study on wild plants used by Tibetan people in Gyirong Valley, Tibet, China

**DOI:** 10.1186/s13002-022-00565-1

**Published:** 2022-11-18

**Authors:** Chang-An Guo, Xiaoyong Ding, Huabin Hu, Yu Zhang, Huizhao Yang, Yuhua Wang

**Affiliations:** 1 Department of Economic Plants and Biotechnology, Yunnan Key Laboratory for Wild Plant Resources, Kunming Institute of Botany, Chinese Academy of Sciences, 132# Lanhei Road, Heilongtan, Kunming, 650201 Yunnan China; 2 University of Chinese Academy of Sciences, Beijing, China; 3 National Centre for Borderland Ethnic Studies in Southwest China, Yunnan University, Kunming, 650091 China; 4 CAS Key Laboratory of Tropical Plant Resources and Sustainable Use, Xishuangbanna Tropical Botanical Garden, Chinese Academy of Sciences, Mengla, 666303 Yunnan China

**Keywords:** Himalayas, Biodiversity hotspots, Tibetan, Traditional knowledge, Environmental conservation

## Abstract

**Background:**

Gyirong Valley known as the “Back Garden of the Himalayas” is located in the core area of the Everest National Nature Reserve. It is also one of the important ports from ancient Tibet to Kathmandu, Nepal, since ancient times. Over the years, the Tibetans of Gyirong had accumulated sufficient traditional knowledge about local plant resources. However, there is almost no comprehensive report available on ethnobotanical knowledge about the local people. The purposes of this study were to (1) conduct a comprehensive study of wild plants used by Tibetan people in Gyirong Valley and record the traditional knowledge associated with wild useful plants, (2) explore the influence of Tibetan traditional culture and economic development on the use of wild plants by local people, and (3) explore the characteristics of traditional knowledge about wild plants of Tibetans in Gyirong.

**Methods:**

Ethnobotanical data were documented through free listings, key informant interviews and semi-structured interviews during fieldwork. The culture importance index and the informant consensus factor index were used as quantitative indices.

**Results:**

In total, 120 informants (61 women and 59 men) and 3333 use reports and 111 wild plant species belonging to 39 families and 81 genera were included. These use reports were then classified into 27 categories belonging to three major categories. The use category that contained the most plant species was edible plants (62), followed by medicinal plants (32) and economic plants (22), and other uses (71). Plants with high CI included *Allium prattii*, *Neopicrorhiza scrophulariiflora*, *Gymnadenia orchidis*, *Rhododendron anthopogon* and *Fritillaria cirrhosa*. Thirty-six species of plants in the catalog of Gyirong and Yadong were the same, but only 17 species were the same in Gyirong and Burang. There were only 11 overlapping species between all the three regions.

**Conclusion:**

Tibetans of Gyirong have rich and unique knowledge about plant use, and wild edible and medicinal plants play an important role in the nutrition and health protection of local people. However, traditional knowledge is slowly being lost and is being hit by modern tourism. In the future, more attention needs to be paid to the important role of traditional knowledge in biodiversity conservation.

## Background

Since antiquity, wild plants have been used for food, medicines, fuel and many other purposes [1, 2]. The collection and consumption of wild plants is an important livelihood part of people living in the underdevelopment area [3–10]. Geography and culture influence the way humans choose to use plants in their behavior and knowledge [11]. However, traditional knowledge is also losing due to the loss of traditional culture and conversion of forest ecosystems to other types of land use, which may be completely lost in future development [12, 13]. Therefore, it is important to record and preserve the traditional plant knowledge associated with plants.

China has a long history of using native plants and a large quantity of recorded knowledge on useful plants [14]. Research on traditional knowledge of wild useful plants is very rich in China, especially in the southwest region [15–18]. The studies here have promoted the recording and protection of traditional plant knowledge, resulting in important guiding significance for the future response to climate change and food and medicine shortages [19].

Tibetans are one of the 56 ethnic minorities in China, mainly living in the Qinghai–Tibet Plateau, with an average altitude of over 4000 m [20, 21]. They have extensive knowledge of wild useful plants [22–26]. As one of the nations living in the Qinghai–Tibet Plateau for generations, Tibetans can adapt to the harsh climate on the plateau, which is inseparable from the use of wild plants, while it is still relatively lacking research. Ethnobotanical research on Tibetans in China is mainly concentrated in Yadong and Purang in the Himalayas, Qinghai, Gansu and Sichuan in western China [22, 27–33]. As for foreign countries, they are mainly concentrated in Nepal, Bhutan and other regions [32, 34–39]. Compared with the huge distribution range of Tibetans, the scope of research on Tibetans is still very narrow; thus, we need more ethnobotanical researches about Tibetans.

Gyirong Valley, known as the “Back Garden of the Himalayas” is located in the core area of the Everest National Nature Reserve to the south of Shigatse City in the Tibet Autonomous Region of China, and the main ethnic group is Tibetan in here [40]. The Gyirong Valley has been an important communication channel between China and South Asian countries since ancient times. It can be said that the Valley has filled half of the history of Tibet [9, 40].

Due to the unique topographical features and history, the Tibetans of Gyirong have accumulated rich traditional knowledge of wild and available plants. This traditional knowledge may have been influenced by Tibetan medicine culture and economic development. The purposes of this study were to (1) conduct a comprehensive study of wild plants used by Tibetan people in Gyirong Valley and record the traditional knowledge associated with wild useful plants, (2) explore the influence of Tibetan traditional culture and economic development on the use of wild plants by local people, and (3) explore the characteristics of traditional knowledge about wild plants of Tibetans in Gyirong.

## Method

### Study area

**Fig. 1:**
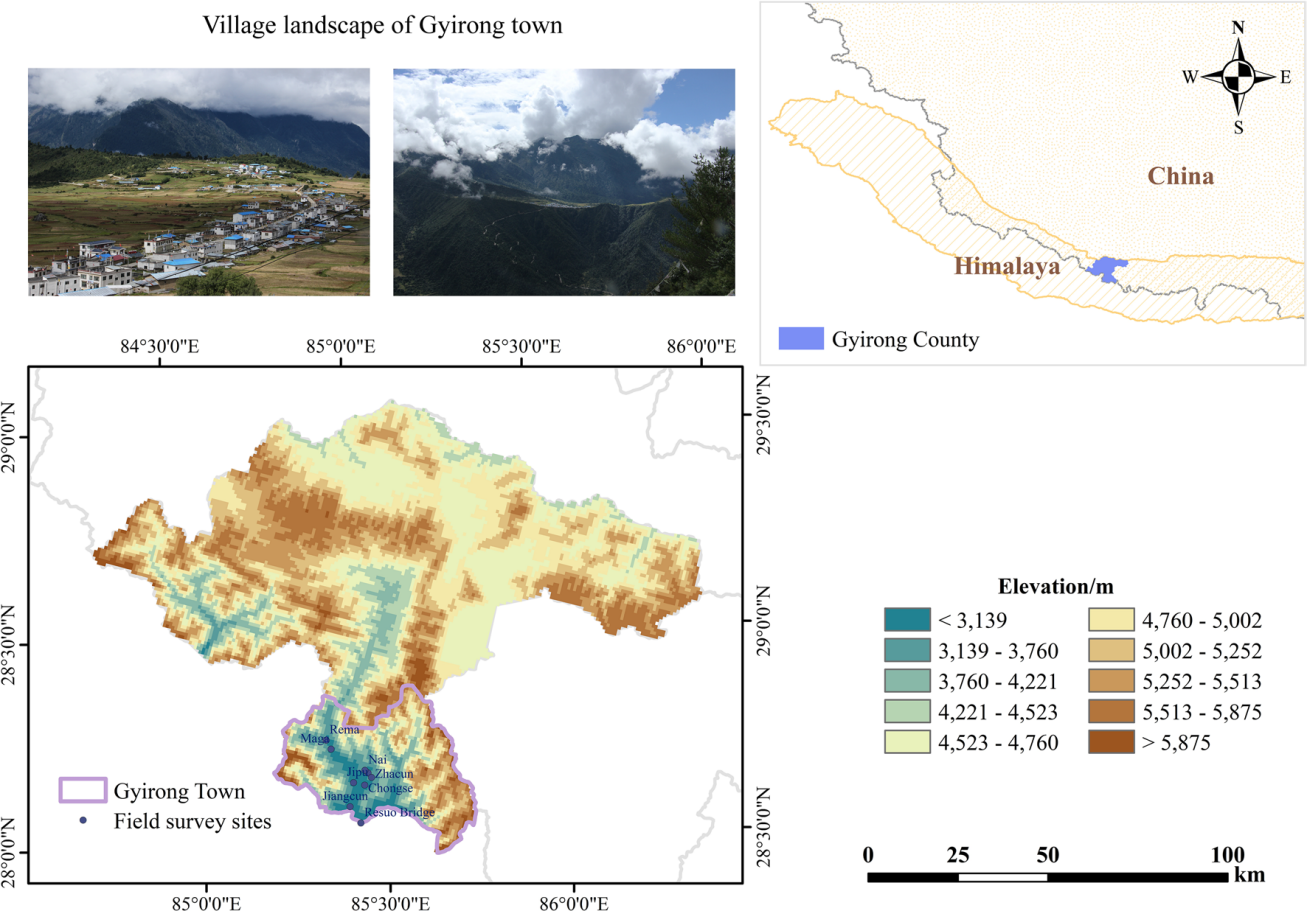
Map showing the location

Gyirong Town is located in the southwest of Shigatse City, Tibet Autonomous Region of China, in the core area of Mount Everest Reserve, and adjacent to Nepal in the south (Fig. 1). The average temperature is 10–13 °C, an annual precipitation of 230–370 mm, and more than 200 frost-free days annually. The vegetation types are mainly mountain coniferous forest and mixed coniferous and broad-leaved forest. It is known as the “back garden of the Himalayas” [23, 40, 41].

The traditional trade between China and Nepal has a long history. The Gyirong Valley has been an important communication channel between China and South Asian countries since ancient times. Since the Han and Tang Dynasties, there have been silk trade traffic routes from mainland China through Tibet, Nepal (known as Nibala in ancient times) and India. Among them, Gyirong is one of the important ports from ancient Tibet to Kathmandu, Nepal [42, 43]. Therefore, Indian and Nepalese styles can still be observed in some temple buildings. Until now, the Tibetans of Gyirong Valley still maintain the traditional customs of transnational trade and intermarriage.

### Tibetans in Gyirong

Tibetans, one of the 56 ethnic groups in China, are divided into three regions according to dialects, namedas Ü-Tsang, Kham and Amdo. The Tibetans in Gyirong Town belong to the Ü-Tsang dialect area [44]. According to local government reports, the livelihood of the local people is dependent on forests and other natural resources apart from agricultural and animal production. Local Tibetans have rich traditional knowledge, such as handicrafts and medicinal plant knowledge [9, 23, 41]. The traditional production practices of the Gyirong Tibetans are agriculture and grazing. The main crops are *Hordeum vulgare*, *Solanum tuberosum*, *Fagopyrum tataricum* and rapeseed. The traditional diet of the Tibetans in Gyirong is mainly tsampa, dairy products, and butter tea.

### Field survey and data collection

The field surveys were conducted between August 2019 and September 2021. Firstly, field study permission was obtained from the local community committee and government authority. Then, we explained our purpose to local governments and requested assistance from them. Because many Tibetans in the study area cannot speak Mandarin fluently, the fieldwork was performed with the assistance of local guides who were employed with the help of local community leaders.

The snowball sampling method was used to select experts who specialize in using plants for healing and make a living using traditional plant knowledge, such as herb dealers, veterinarians and traditional healers. A randomized household interview method was used to select other informants. Data were collected through individual semi-structured interviews conducted from local 120 informants, which constitutes the classic method in ethnobiology (Table 1). All interviews were conducted in the Tibetan language, which was translated into Mandarin by local guides. All field studies were conducted with the consent of informants. The wild useful plants and related traditional knowledge were documented. The following are the questions in the semi-structured interviews:

1. Would you mind listing some wild plants you have used in the Tibetan language?
2. How to use these plants, food, medicine, fodder or other purposes?
3. Which plant parts were used, roots, stems, leaves or other parts?
4. Why do you use this species?
5. What time do you collect this plant?

**Table 1:** Characteristics of informants

Characteristics	Number	Percentage
*Communities*		
Chongse	5	4.2%
Jiangcun	9	7.5%
Jipu	16	13.3%
Langjiu	9	7.5%
Maga	20	16.7%
Nai	22	18.3%
Rema	18	15.0%
Resuo bridge	3	2.5%
Zhacun	18	15.0%
*Gender*		
Female	61	50.8%
Male	59	49.2%
*Age*		
Below 20	5	4.2%
20–29	12	10.0%
30–39	19	15.8%
40–49	27	22.5%
50–59	26	21.7%
60–69	21	17.5%
70–79	7	5.8%
Above 80	3	2.5%

The questions were designed to collect data on the (1) vernacular names of the plants, (2) use categories, (3) used parts, and (4) preparation and administration methods.

The specimens were collected from the field of the survey with the help of the key informants and all materials were labeled with numbers and local names. We use Chinese pinyin to encode local names. Photographs of each plant were taken. All specimens were kept in the herbarium of the Kunming Institute of Botany (KUN). The Flora of China was used to help identify the plants [45], and The Plants of the World Online was used to ensure the Latin name of the plants [46].

### Data analysis

We adopted the cultural important index (CII) and the informant consensus factor index (FIC) as ethnobotanical indices. All information about wild used plants was organized into a “use report” list consisting of three parts: informant, plant and use category [47, 48]. The criteria for the use categories of plants mainly refer to The Economic Botany Data Collection Standard (EBDCS) [48].

The cultural important index (CII) was the sum of the proportion of informants that mentioned each of the use categories for a given species [49]. In other words, CII represents the diversity of plant uses and the degree of recognition of information sources for each use category. This index is used to quantitatively evaluate the importance of a certain plant to Tibetan of Gyirong from the perspective of comprehensive value.

The calculation formula is as follows:

$${\text{CII}} = \mathop \sum \limits_{U = u1}^{{{\text{uNC}}}} \mathop \sum \limits_{i = i1}^{{{\text{iN}}}} \frac{{{\text{URui}}}}{N}$$

NC is the total number of use categories and N is the total number of informants. CII ranges between 0 and the number of all use categories, and the index is greater than 1 if the number of mentions of the plant is greater than the total number of informants. A higher CII value indicated the multiple uses of a species and a higher degree of recognition

The informant consensus factor index (FIC) was developed by Trotter [50]. FIC was used to evaluate the degree of consensus among the population about how to treat a particular disease. The calculation formula is as follows:

$${\text{FIC}} = \frac{{{\text{Nur}} - {\text{Nt}}}}{{{\text{Nur}} - 1}}$$

where Nur is the number of use reports from the informants for a particular disease and Nt is the total number of plant species used to treat the disease. The FIC values range between 0 and 1. A higher FIC means that different herbalists have a higher consensus on the plant species used for certain use categories.

## Results

### The diversity of wild plants used by locals

Tibetan people living in Gyirong Valley use a variety of wild plants. A total of 111 wild plant species, including scientific names, vernacular names, usage, used part, the number of UR and CII. All the information of these plants is given in Table 2.

**Table 2:** List of plants used by the Tibetan people in Gyirong

Botanical taxon	Botanical family	Local name(s)	Voucher	Parts used	Local use (No. of URs) and preparation	UR	CI
*Chenopodium album* L	Amaranthaceae	Lei1; niu1; niu1-che1-ma1	QTB-JL-42	Leaves	Food: vegetable (14), used to make bun or fry	14	0.111
*Allium chrysanthum* Regel	Amaryllidaceae	Guo1-ba1	QTB-JL-21	Roots	Food: vegetable (20), used to make bun or fry; seasoning (7), cook it with pork and potatoes	27	0.214
*Allium fasciculatum* Rendle	Amaryllidaceae	Ou1-ga1	QTP-EBT-3050	Aerial parts	Food: vegetable (10), used to make bun or fry; seasoning (7), cook it with pork and potatoes	17	0.135
*Allium prattii* C.H.Wright	Amaryllidaceae	Ru1-ba1; ri1-guo1	QTP-EBT-3009	Aerial parts	Food: vegetable (126), used to make bun or fry; seasoning (9), cook it with pork and potatoes	141	1.119
*Allium przewalskianum* Regel	Amaryllidaceae	Ri1-guo1; zen1-bu1	QTP-EBT-3200	Leaves	Food: seasoning (19), as part of the dip; economic (1), be sold in stores	20	0.159
*Allium wallichii* Kunth	Amaryllidaceae	Ru1-guo1	QTB-JL-55	Aerial parts	Food: seasoning (6), cook it with pork and potatoes or as part of the dip	6	0.048
*Angelica sinensis* (Oliv.) Diels	Apiaceae	Dang1-gui1	QTB-JL-41	Roots	Medicine: tonic (3), cook it with meat	3	0.024
*Carum carvi* L	Apiaceae	Guo1-nie1	QTB-JL-63	Leaves; seeds	Food: vegetable (25), the leaves are cooked with other ingredients; seasoning (35), use seeds to make a dip or a sausage; medicine: gastralgia (5), seed soak in water	65	0.516
*Chaerophyllum villosum* Wall. ex DC	Apiaceae	Da1-ga1-li1	QTB-JL-20	Leaves	Economic (3), be sold in stores; food: seasoning (1), cook its leaves with meat; fodder (1): the leaves are used to feed cattle	5	0.040
*Heracleum candicans* Wall. ex DC	Apiaceae	Jiong4-wa1-dong1-bu4	QTB-JL-43	Aerial parts	Medicine: headache (1); soak in water	1	0.008
*Aralia* sp.	Araliaceae	Jia1-ra3-cei3-ma1	QTB-JPG-10	Leaves	Food: vegetable (2), cook vegetable	2	0.016
*Aralia tibetana* G.Hoo	Araliaceae	Dai1-ga1-ni1	QTB-JL-46	Whole plant	Fodder (5): the leaves are used to feed cattle	5	0.040
*Panax pseudoginseng* Wall	Araliaceae	San1-jing1	QTP-EBT-3084	Roots	Medicine: tonic (30), soak in water; economic (25): be sold in stores	55	0.437
*Polygonatum cirrhifolium* (Wall.) Royle	Asparagaceae	ra3-mu1-xia3-jia1	QTB-JL-1	Aerial parts; roots	Food: vegetable (26), cook vegetable; economic (8), be sold in store; medicine: nephropathy (11), roots soak in water or make soup	35	0.278
*Polygonatum sibiricum* Redouté	Asparagaceae	Rang3-ma1-xia-jia1	QTB-JL-26	Roots; aerial parts	Medicine: tonic (15), roots soak in water or make soup; food: vegetable (45), cook vegetable; economic (12), be sold in store; fodder (3): used to feed cattle	75	0.595
*Artemisia calophylla* Pamp	Asteraceae	Bang1-ma1	QTB-JL-50	Aerial parts	Ritual use (4), used to burn in incense burner; medicine: rheumatism (72): used it to sweat steaming or leaves soak in water to drink	76	0.603
*Artemisia japonica* Thunb	Asteraceae	Kang1-ba1	QTB-JL-59	Aerial parts	Ritual use (60), used to burn in incense burner; medicine: detoxification (23), soak in water; economic (4): be sold in store	87	0.690
*Artemisia younghusbandii* J. R. Drumm. ex Pamp	Asteraceae	Sang1-kang3-ba1	QTB-JL-49	Aerial parts	Ritual use (4), used to burn in incense burner; medicine: rheumatism (4), used it to sweat steaming or leaves soak in water to drink	8	0.063
*Galinsoga parviflora* Cav	Asteraceae	Cuo1-ma1	QTP-JPG-6	Whole plants	Fodder (1): used to feed cattle	1	0.008
*Leontopodium souliei* Beauverd	Asteraceae	Ba1-wa1	EBT-PL-99	Leaves	Tool (2), used it to start a fire	2	0.016
*Saussurea tridactyla* Sch.Bip. ex Hook.f	Asteraceae	Gang3-la1-mei3-duo3	QTB-JL-66	Whole plants	Economic (36), be sold in store; medicine: arthrophlogosis (46): soaked in water	82	0.651
*Taraxacum sikkimense* Hand.-Mazz	Asteraceae	se4-ji4-mei3-duo3	QTB-JL-110	Whole plant	Medicine: endocrine (3), soaked in water; economic (2), be sold in store	5	0.040
*Impatiens bicornuta* Wall	Balsaminaceae	Po1-zi1	QTB-JL-73	Seeds	Varnish (12), used to polish furniture	12	0.095
*Impatiens falcifer* Hook.f	Balsaminaceae	Po1-zi1	QTB-JL-15	Seeds	Varnish (14), used to polish furniture	14	0.111
*Impatiens scabrida* DC	Balsaminaceae	Po1-zi1	QTB-JL-70	Seeds	Varnish (13), used to polish furniture	13	0.103
*Impatiens sulcata* Wall	Balsaminaceae	Po1-zi1	QTB-JL-62	Seeds	Varnish (11), used to polish furniture	11	0.087
*Berberis angulosa* Wall. ex Hook.f. & Thomson	Berberidaceae	jiu1-bo1; jiu1-le1-bu1	QTB-JL-113	Leaves; fruits; branches	Medicine: diarrhea (1), soaked in water; food: fruit (7), raw; fuelwood (2): used to burn	10	0.079
*Berberis aristata* DC	Berberidaceae	jiu1-lu1-xin1	QTB-JL-28	Fruits; branches	Fuelwood (3), used to burn; food: fruit (2), raw	5	0.040
*Berberis xanthophlaea* Ahrendt	Berberidaceae	giu1-lu1; giu1-le1-bu1; gei1-lu1-mi3-xia4	QTB-JL-27	Barks; fruits	Dyes (10), used to dye wool yellow; food: fruit (4), raw	14	0.111
*Stauntonia angustifolia* (Wall.) R.Br. ex Wall	Berberidaceae	pa1-ji1	QTP-JPG-2	Fruits	Food: fruit (2), raw	2	0.016
*Betula utilis* D.Don	Betulaceae	da4-ge1-ba1	QTB-JL-7	Burls; branches; stems	Medicine: diabetes (8), soak in water; economic (2), be sold in store; fuelwood (14), used to burn; craft (7), used to make Tibetan traditional wooden bowls; ritual use (8), used to burn in incense burner; tool (6), used to make cooking utensils	37	0.294
*Onosma hookeri* C.B. Clarke	Boraginaceae	Guo1-mu1-mu1-zi1	QTP-EBT-3052	Roots	Medicine: hair follicle (4), soaked in canola oil and apply to the head; eczema (4); ritual use (7), used to burn in incense burner; economic (1), be sold in store	16	0.127
*Capsella bursa-pastoris* (L.) Medik	Brassicaceae	Du1-yang1	QTB-JL-34	Aerial parts	Food: vegetable (8), cooked vegetable	8	0.063
*Thlaspi arvense* L	Brassicaceae	Mang3-ru1	QTB-JL-35	Leaves	Food: vegetable (10), cooked vegetable	10	0.079
*Cannabis sativa* L	Cannabaceae	Si1-ma1	QTB-JL-78	Barks	Tool (8); fodder (4), used to feed cattle	12	0.095
*Dipsacus asper* Wall. ex C.B. Clarke	Caprifoliaceae	Lang1-zhu1-ma1	QTP-EBT-3053	Aerial parts	Fodder (1), used to feed cattle	1	0.008
*Lonicera* sp.	Caprifoliaceae	se4-le4-qin1-mei3-duo3	EBT-PL-42	Flowers	Economic (2), be sold in store	2	0.016
*Nardostachys jatamansi* (D.Don) DC	Caprifoliaceae	Bang1-bu4	QTB-JL-123	Roots	Ritual use (87), used to burn in incense burner; economic (1), be sold in store; medicine: relieving cough and asthma (4), soaked in water	92	0.730
*Coriaria terminalis* Hemsl	Coriariaceae	da1-lu1	QTP-EBT-3005	Fruits	Food: fruit (1), raw	1	0.008
*Rhodiola himalensis* (D. Don) S.H. Fu	Crassulaceae	suo3-la1-ma3-bu4	QTB-JL-124	Stems	Medicine: tonic (20), hypertension (28), soaked in water; economic (19), be sold in store; ritual use (3), used to burn in incense burner	70	0.556
*Cyclanthera pedata* (L.) Schrad	Cucurbitaceae	ra3-ru1	QTB-JPG-12	Fruits	Food: vegetable (7), cooked vegetable	7	0.056
*Herpetospermum pedunculosum* (Ser.) C.B. Clarke	Cucurbitaceae	sei1-lei1; sei1-lei1-mei3-duo3	QTB-JL-22	Flowers; fruits	Medicine: diarrhea (12), powder; veterinary medicine: diarrhea (3), powder	15	0.119
*Solena heterophylla* Lour	Cucurbitaceae	ma1-ma1-dong4-cei1	QTB-JL-80	Fruits	Food: fruit (6), raw	6	0.048
*Trichosanthes lepiniana* (Naudin) Cogn	Cucurbitaceae	ka1-ge1-di1	QTB-JL-24	Seeds	Medicine: fever (1), poder; economic (1), be sold in store	2	0.016
*Juniperus indica* Bertol	Cupressaceae	xiu1-bai1	QTB-JL-57	Branches; stems	Ritual use (67), used to burn in incense burner; fuelwood (7), used to burn; craft (6)	80	0.635
*Juniperus tibetica* Kom	Cupressaceae	xiu1-bo1	QTB-JL-64	Branches; stems	Ritual use (38), used to burn in incense burner; fuelwood (22), used to burn; craft (20), used to make Tibetan traditional wooden bowls; food: fruit (2), raw	82	0.651
*Pteridium aquilinum* var. *latiusculum* (Desv.) Underw. ex A. Heller	Dennstaedtiaceae	da1; da1-gu1; dai1-ga1; da1-li1	QTB-JL-10	Leaves	Food: cooked vegetable (86)	86	0.683
*Elaeagnus umbellata* Thunb	Elaeagnaceae	ra1-lu1	QTB-JL-18	Fruits	Food: fruit (43), raw	43	0.341
*Hippophae salicifolia* D.Don	Elaeagnaceae	da1-ru1	QTB-JL-16	Fruits; branches	Food: fruit (21), raw; seasoning (14), fruit juice is used as a substitute for vinegar; medicine: arthrophlogosis (3), the juice is used to smear the joints; fuelwood (1), used to burn	39	0.310
*Rhododendron anthopogon* D. Don	Ericaceae	po1-lu1	QTB-JL-115	Branches; flowers	Ritual use (91), used to burn in incense burner; fuelwood (1), used to burn; medicine: eyes ache (4), arthrophlogosis (4), flowers are used to soak water; beverage (6), soak in water; economic (4), be sold in store	110	0.873
*Rhododendron arboreum* Sm	Ericaceae	mei3-duo1	QTB-JL-30	Branches; stems	Fuelwood (7), used to burn; craft (9), used to make Tibetan traditional wooden bowls	16	0.127
*Rhododendron lepidotum* Wall. ex G. Don	Ericaceae	su1-lu1	QTB-JL-114	Branches	Ritual use (15), used to burn incense burner	15	0.119
*Euphorbia micractina* Boiss	Euphorbiaceae	ta3-lu1-ma1	QTB-JL-85	Leaves	Medicine: poisons (2)	2	0.016
*Cicer microphyllum* Benth	Fabaceae	pu3-gui3	EBT-PL-13	Fruits	Food: fruit (2), raw	2	0.016
*Quercus semecarpifolia* Sm	Fagaceae	bai1-luo4	QTB-JL-25	Stems; branches; leaves	Ritual use (2), used to burn in incense burner; craft (15), used to make Tibetan traditional wooden bowls; fuelwood (48); fodder (5), used to feed cattle; food: starch (4), cooked fruit	74	0.587
Gentiana veitchiorum Hemsl	Gentianaceae	bang1-jie1-mei3-duo3	QTP-EBT-3024	Whole plant	Medicine: fever (18), soaked in water	18	0.143
*Swertia cordata* (Wall. ex G. Don) C.B. Clarke	Gentianaceae	di1-ge1-da1	QTP-EBT-3111	Aerial parts	Medicine: fever (10), soak in water; economic (2); veterinary medicine (1), soak in water	13	0.103
*Isoetes hypsophila* Hand.-Mazz	Isoetaceae	pa1-xia4	QTP-JPG-3	Leaves	Food: vegetable (25), cooked vegetable	25	0.198
*Juglans regia* L	Juglandaceae	da1-ba1	QTB-JL-88	Fruits; stems; branches	Dyes (23), pericarp used to dye the container black; ritual use (9), used to burn in incense burner; food: fruit (9), raw; craft (30), used to make Tibetan traditional wooden bowls; fuelwood (12), used to burn	83	0.659
*Elsholtzia fruticosa* (D.Don) Rehder	Lamiaceae	ma1-zei1	QTB-JL-48	Aerial parts	Ritual use (12), used to burn in incense burner; fuelwood (5), used to burn	17	0.135
*Nepeta densiflora* Kar. & Kir	Lamiaceae	pi1-ba4	QTP-EBT-3060	Aerial parts	Fodder (3), used to feed cattle	3	0.024
*Fritillaria cirrhosa* D.Don	Liliaceae	bai1-mu4	QTP-EBT-3012	Bulbs	Medicine: tonic (40), stew or soak in water; cold (20); economic (45), be sold in store; veterinary medicine (1), soak in water; food: fruit (2), raw	108	0.857
*Malva verticillata* L	Malvaceae	jiang4-ba1-la1-mu1	QTB-JL-36	Roots; leaves	Food: vegetable (25), cooked vegetable	25	0.198
*Paris polyphylla* Sm	Melanthiaceae	bo1-luo3	QTP-EBT-3085	Arieal parts	Ritual use (11), used to burn in incense burner; medicine: stomachache, soaked in water or wine (10); economic (2), be sold in store; vegetable (28), cooked vegetable	51	0.405
*Gastrodia elata* Blume	Orchidaceae	tian3-ma3	QTP-JPG-3292	Roots	Economic (29), be sold in store; medicine: headache (6), cardiopathy (40), slice and soak in water or stew; food: vegetable (2), used to make soup with chicken	77	0.611
*Gymnadenia orchidis* Lindl	Orchidaceae	wang1-bu1-la1-ba1	QTB-JL-56	Roots	Medicine: pulmonary disease (5), soak in water or wine; burn (32), acne (38), used to daub affected area; economic (31); ritual use (10), raw materials for making Tibetan incense	116	0.921
*Phytolacca acinosa* Roxb	Phytolaccaceae	Wo1-yang1	QTB-JL-84	Leaves	Food: vegetable (13), cooked vegetable	13	0.103
*Larix potaninii* var. *himalaica* (W.C.Cheng & L.K.Fu) Farjon & Silba	Pinaceae	Long3-xin1	QTP-JPG-7	Branches; stems	Fuelwood (4), used to burn; craft (9), used to make Tibetan traditional wooden bowls	13	0.103
*Pinus wallichiana* A.B.Jacks	Pinaceae	Nong1-xin1; tang3-xin1	QTB-JL-39	Branches; stems	Fuelwood (44), used to burn; craft (7), used to make Tibetan traditional wooden bowls; ritual use (11), used to burn in incense burner; food: vegetable (7), cooked vegetable	73	0.579
*Neopicrorhiza scrophulariiflora* (Pennell) D.Y.Hong	Plantaginaceae	di1-da1; hong1-lei1	QTB-JL-67	Roots	Medicine: cold and fever (107), soaked in water; economic (18), be sold in store; veterinary medicine: fever (3), soak in water	128	1.016
*Plantago asiatica* L	Plantaginaceae	wo1-ma1-ka1	QTP-EBT-3117	Aerial parts	Medicine: hypertension (4), soaked in water; food: vegetable (3), cooked vegetable	7	0.056
*Plantago asiatica* subsp. *densiflora* (J.Z.Liu) Z.Y.Li	Plantaginaceae	ou3-ma1-ka3	QTB-JL-12	Leaves; roots	Medicine: hypertension (2), soaked in water; food: vegetable (3), cooked vegetable	5	0.040
*Avena fatua* L	Poaceae	sei1-za1-ba1	QTB-JPG-11	Aerial parts	Fodder (1), used to feed cattle	1	0.008
*Fargesia* sp.	Poaceae	niu1-dong1	QTB-JL-118	Stems	Economic (1), be sold in store; food: vegetable (66), cooked vegetable; craft (19), used to make bamboo plaits; ritual use (6), used to burn in incense burner; fuelwood (2), used to burn; fodder (7), used to feed cattle	101	0.802
*Poaceae* sp.	Poaceae	zang4-ong1-bu4	QTP-JPG-8	Whole plant	Fodder (7), used to feed cattle	7	0.056
*Fagopyrum esculentum* Moench	Polygonaceae	bai1-bi1-ya1	QTB-JL-60	Arieal parts	Fodder (3), used to feed cattle	3	0.024
*Fallopia denticulata* (C.C.Huang) Holub	Polygonaceae	a1-lang1-ba1-lang1	QTB-JL-122	Aerial parts; roots	Fodder (2), used to feed cattle; medicine: diarrhea (5), hair follicle (2), soak in water	9	0.071
*Koenigia tortuosa* (D.Don) T.M.Schust. & Reveal	Polygonaceae	nia1-luo1	QTB-JL-4	Aerial parts; stems	Dye (6), used to dye wooden bowls or clothes yellow; fodder (8), used to feed cattle; food: fruit (4), raw	18	0.143
Pteroxygonum denticulatum (C.C.Huang) T.M.Schust. & Reveal	Polygonaceae	ren3-bu1	QTB-JL-45	Aerial parts	Fodder (2), used to feed cattle	2	0.016
*Rheum australe* D. Don	Polygonaceae	qu1-wa1; jiong1	QTB-JL-3	Stems; roots	Fruit (17), raw eat tender stem; dye (53), used to dye wooden bowls or clothes yellow	70	0.556
*Rumex nepalensis* Spreng	Polygonaceae	xiu1-ma1	EBT-PL-86	Aerial parts	Fodder (2), used to feed cattle	2	0.016
*Aconitum jilongense* W.T.Wang & L.Q.Li	Ranunculaceae	beng3-ga1	QTB-JPG-1	Roots	Medicine: diarrhea (24), soak in water	24	0.190
*Clematis rehderiana* Craib	Ranunculaceae	ba1-ji1-ma1	EBT-PL-84	Leaves	Food: vegetable (2), cooked vegetable	2	0.016
*Delphinium kamaonense* Huth	Ranunculaceae	jia1-bei1-mei1-duo1	QTB-JL-37	Aerial parts	Fodder (1), used to feed cattle	1	0.008
*Eriocapitella rivularis* (Buch.-Ham. ex DC.) Christenh. & Byng	Ranunculaceae	cei1-di1-ma1	QTB-JPG-9	Aerial parts	Fodder (1), used to feed cattle	1	0.008
*Gymnaconitum gymnandrum* (Maxim.) Wei Wang & Z.D.Chen	Ranunculaceae	zen1-du1; du3-wa1-ten3-du1	QTP-EBT-3097	Roots	Medicine: poisons (16), rheumatism (16), soaked in water and apply to the affected area; economic (4), be sold in store	36	0.286
*Berchemia flavescens* (Wall.) Wall. ex Brongn	Rhamnaceae	bo1-ge1-da4	QTB-JL-93	Fruits	Food: fruit (45), raw	45	0.357
*Argentina anserina* (L.) Rydb	Rosaceae	chu1-ma1	QTP-EBT-3055	Roots	Food: starch (60), cooked and eat with yogurt or rice	60	0.476
*Chaenomeles thibetica* T.T.Yu	Rosaceae	bai1-la1	QTB-JL-109	Fruits	Food: fruit (19), raw; fuelwood (3), used to burn	22	0.175
*Fragaria nubicola* (Lindl. ex Hook.f.) Lacaita	Rosaceae	long1-mei1; sei1-duo1-zhe3-xin1	QTB-JL-9	Fruits; stems	Fruit (83), raw; ritual use (3), used to burn in incense burner	86	0.683
*Griffitharia vestita* (Wall. ex G.Don) Rushforth	Rosaceae	na1-zi1	QTB-JL-5	Fruits; branches	Food: fruit (55), raw; fuelwood (1), used to burn; ritual use (2), used to burn in incense burner	58	0.460
*Prinsepia utilis* Royle	Rosaceae	bu1-long1-che4-mang1	QTB-JL-38	Seeds	Economic (9), be sold in store	9	0.071
*Prunus holosericea* (Batal.) Kost	Rosaceae	a1-lu1-ba3-lu1	QTB-JL-91	Fruits	Food: fruit (6), raw	6	0.048
*Prunus mira* Koehne	Rosaceae	kang3-bu4	QTB-JL-69	Fruits	Food: fruit (53), raw	53	0.421
*Rosa macrophylla* Lindl	Rosaceae	sei1-duo1	QTB-JL-29	Branches; fruits	Fuelwood (3), used to burn; food: fruit (11), raw	14	0.111
*Rosa sericea Lindl*	Rosaceae	gu1-jiu1-ma1; gun1-zhong1	QTB-JL-17	Fruits; branches	Food: fruit (91), raw; fuelwood (1), used to burn; medicine: digestion (1), raw	94	0.746
*Rubus aurantiacus* Focke ex Sarg	Rosaceae	ni1-na1	QTB-JL-14	Fruits	Food: fruit (6), raw	6	0.048
*Rubus austrotibetanus* T.T.Yu & L.T.Lu	Rosaceae	nia1-lang1	QTB-JL-82	Fruits	Food: fruit (55), raw	55	0.437
*Rubus biflorus* Buch.-Ham. ex Sm	Rosaceae	nie1-sen1; nia1-lang1	QTB-JL-83	Fruits	Food: fruit (9), raw	9	0.071
*Rubus niveus* Thunb	Rosaceae	nia1-lang2	QTB-JL-13	Fruits	Food: fruit (68), raw	68	0.540
*Thomsonaria ochracea* (Hand.-Mazz.) Rushforth	Rosaceae	ca1-le1-ba1	QTB-JL-92	Branches	Fuelwood (17), used to burn; tool (3), used to make handle; ritual use (1), used to burn in incense burner	21	0.167
*Zanthoxylum bungeanum* Maxim	Rutaceae	ei1-ma1	QTB-JL-8	Fruits; seeds	Food: seasoning (83), vegetable (14), cooked it with meat; economic (3), be sold in store; medicine: endocrine (3), raw or soak in water; fuelwood (1), used to burn	104	0.825
*Salix babylonica* f. *babylonica*	Salicaceae	jiang1-ma1	QTB-JL-108	Branches	Fodder (5), used to feed cattle; fuelwood (3), used to burn; ritual use (3), used to burn in incense burner	11	0.087
*Salix trichocarpa* C.F. Fang	Salicaceae	lang1-ma1	QTB-JL-47	Branches; flowers; stems	Fuelwood (15), used to burn; ritual use (14), used to burn in incense burner; fodder (1), used to feed cattle; craft (3), used to make wooden bowl; food: vegetable (2), flower buds can be fried and eaten	35	0.278
*Schisandra elongata* (Blume) Baill	Schisandraceae	gong1-zhu1	QTB-JL-117	Fruits	Food: fruit (7), raw	7	0.056
*Tamarix chinensis* Lour	Tamaricaceae	ong1-bu4	QTB-JL-18	Branches	Ritual use (1), burned to sacrifice to the dead	1	0.008
*Taxus wallichiana* Zucc	Taxaceae	sei1-ge1-xia4	QTB-JL-31	Branches; fruits	Fuelwood (11), used to burn; food: fruit (5), raw	16	0.127
*Urtica ardens* Link	Urticaceae	suo3-wa1	QTP-JPG-5	Leaves	Food: vegetable (36), cooked vegetable	36	0.286
*Urtica urens* L	Urticaceae	suo3-wa1	QTP-JPG-4	Leaves	Food: vegetable (23), cooked vegetable	23	0.183
*Viburnum cotinifolium* D. Don	Viburnaceae	gei1-jiu1-ma1	QTB-JL-51	Fruits	Food: fruit (4), raw	4	0.032
*Viburnum nervosum* D. Don	Viburnaceae	ka3-la1-suo1	QTB-JL-102	Fruits	Food: fruit (6), raw	6	0.048

**Fig. 2:**
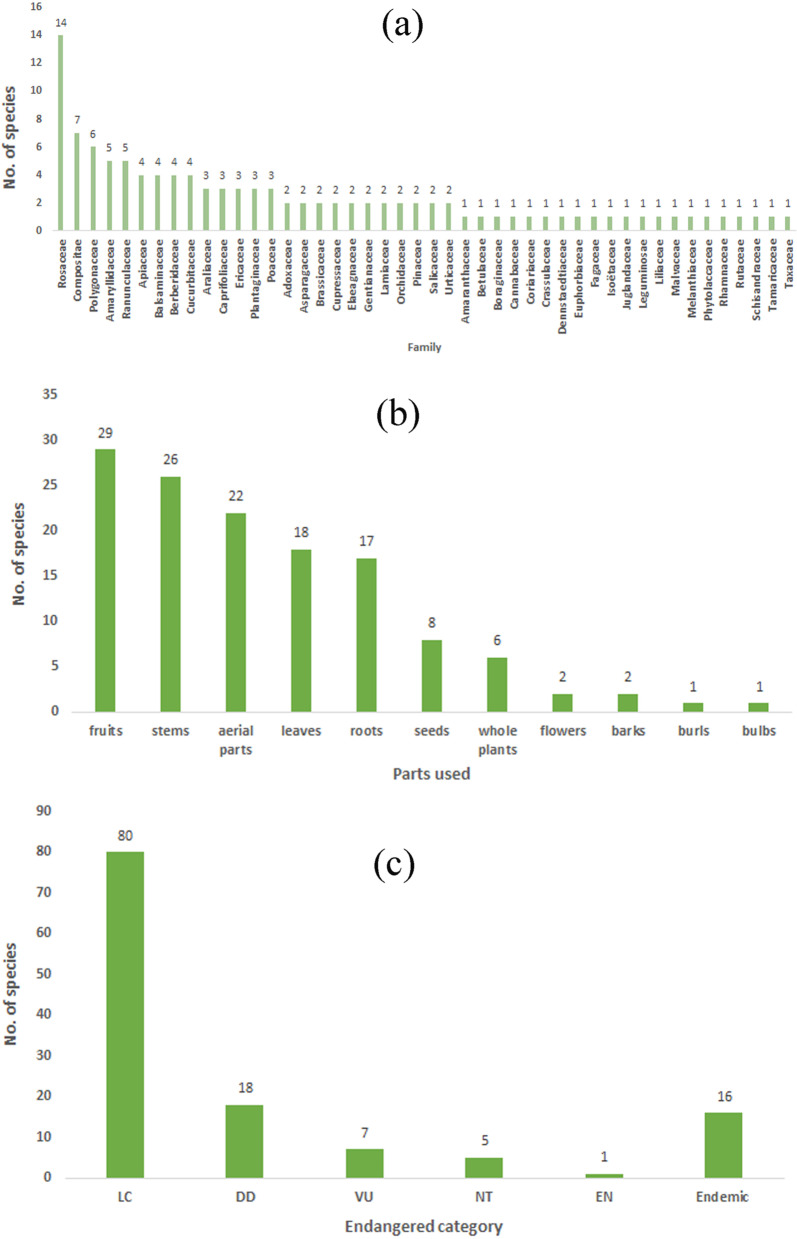
Diversity of wild plants used by locals. **a** diversity of families; **b** diversity of used parts; **c** threatened species,  LC = least concerned, DD = data deficient, VU = vulnerable, NT = near-threatened, EN = endangered

The taxonomic types of wild plants used by Tibetan people included angiosperms (104 species), gymnosperms (5) and ferns (2). These plants belong to 39 families and 81 genera. Rosaceae (14) was the most represented family, followed by Compositae (7) and Polygonaceae (6) (Fig. 2a). The life forms of these plants are mostly herbs (64), followed by trees (19), shrubs (19) and vines (8).

The use parts are diverse, including fruits, roots, leaves, stems, whole plants, aerial parts, bulbs, barks, seeds, pericarps and tubers. The most used parts are fruits (29 species), followed by stems (25) and roots (17) (Fig. 2b).

Among all wild useful plants, there are 13 endangered plant species, of which *Neopicrorhiza scrophulariiflora* is endangered (EN) level. Seven species are vulnerable (VU) levels. Five species are near-threatened (NT) levels. Among these 13 species, three species are economic plants, and the others were edible and medicinal plants. In addition, there are 16 species endemic to China among all useful plants [51] (Fig. 2c).

### The diversity of wild edible plants

**Fig. 3:**
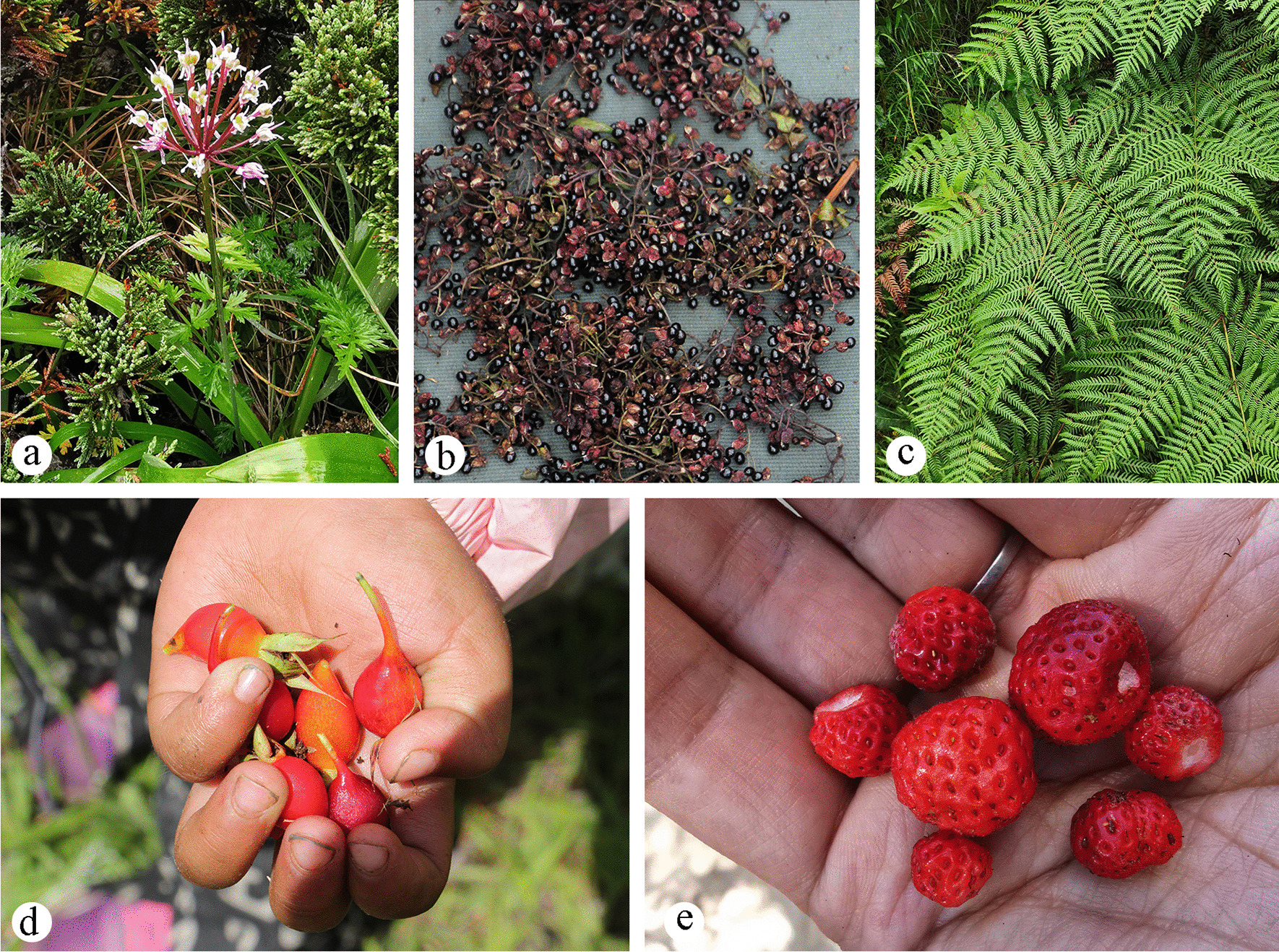
The most frequently reported edible plants **a** *Allium prattii*; **b** *Zanthoxylum bungeanum*; **c** *Rosa sericea*; **d** *Pteridium aquilinum* var. *latiusculum*; **e** *Fragaria nubicola*

Wild edible plants (WEPs) were the most frequently used in all categories with 62 edible species belonging to 33 families, and the main used parts of WEPs are fruits and stems. The use categories of WEPs include fruits, vegetables, seasonings, food substitutes and tea substitutes (Table 3). Among these, fruit is the most frequently used (30 species), followed by wild vegetables (27). The most frequently reported species were *Allium prattii* (135), followed by *Zanthoxylum bungeanum* (97), *Rosa sericea* (91), *Pteridium aquilinum* var. *latiusculum* (86) and *Fragaria nubicola* (83) (Fig. 3).

**Table 3:** Use categories

Local use	Secondary use categories	Ns	URs
Edible plants		62	1533
	Fruits	30	617
	Seasonings	9	173
	Vegetables	27	677
	Beverages	1	6
	Starches	1	60
Medicinal plants		32	614
	Poison	2	18
	Inflammation	1	32
	Poisonings	1	23
	Infections	2	11
	Digestive system disorders	7	53
	Respiratory system disorders	4	134
	Nutritional disorders	5	108
	Endocrine system disorders	2	6
	Muscular–skeletal system disorders	5	142
	Genitourinary system disorders	2	19
	Skin disorders	4	48
	Veterinary medicine	4	8
	Nervous system disorders	2	7
	Circulatory system disorders	4	74
	Eyes	1	4
Economic plants	Improve livelihoods	22	261
Other use		69	1037
	Tools	5	21
	Crafts	10	123
	Dyes	4	87
	Fodders	20	63
	Fuelwoods	19	215
	Ritual plants	24	528

### The diversity of wild medicinal plants and evaluation of medicinal plants based on FIC

**Fig. 4:**
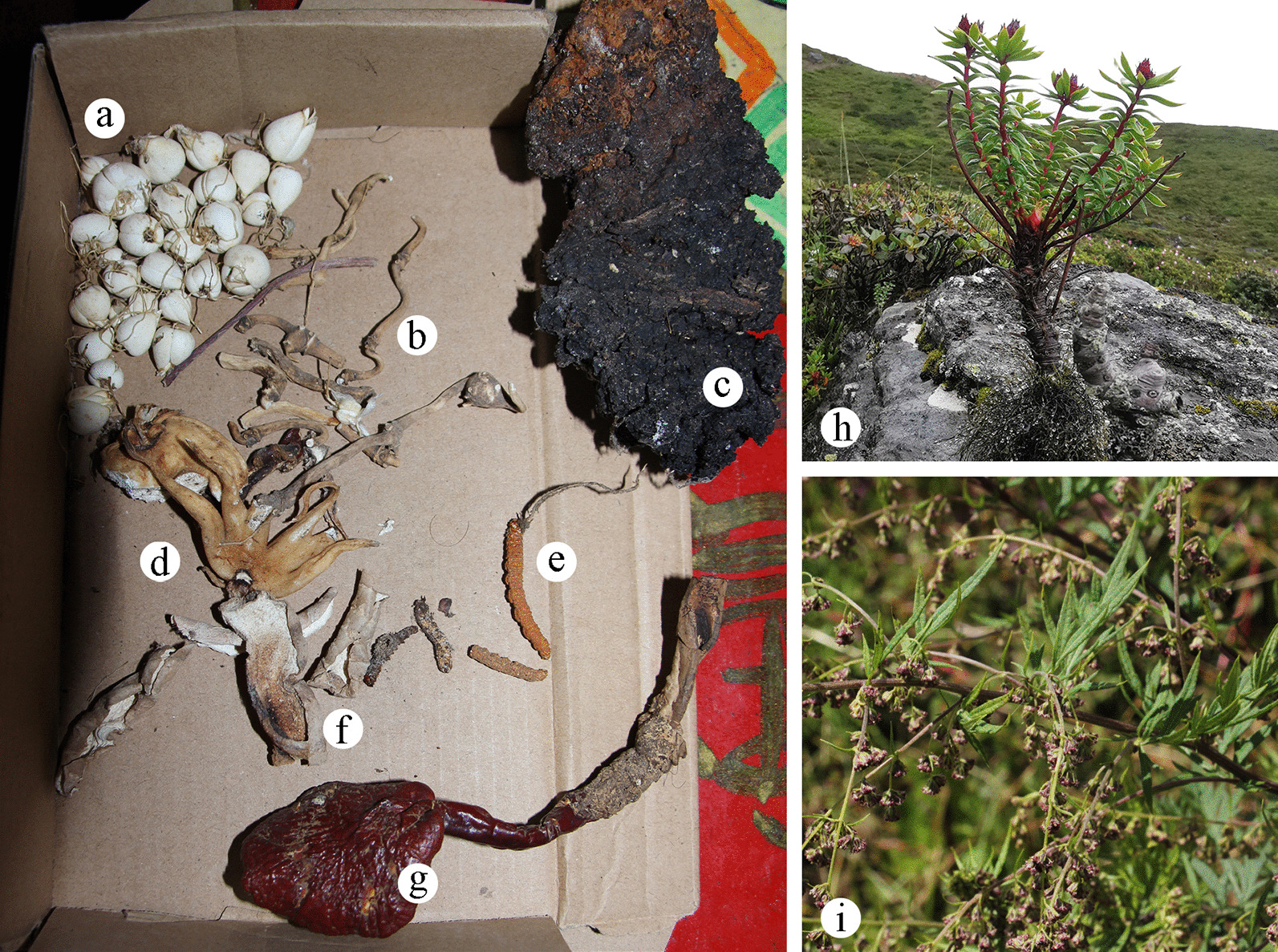
Some medicinal plants in the study area. **a** *Fritillaria cirrhosa*; **b** *Panax pseudoginseng*; **c** *Betula utilis*; **d** *Gymnadenia orchidis*; **e** *Ophiocordyceps* sp.; **f** *Gastrodia elata*; **g** *Ganoderma* sp.; **h** *Rhodiola himalensis*; **i** *Artemisia calophylla*

Wild medicinal plants are the second largest category of wild plants used by the Tibetan people of Gyirong town. These plants belong to 22 families and have been documented to treat 15 different types of human diseases. The most frequently mentioned were muscular–skeletal system disorders, followed by respiratory system disorders (Table 3). The family with the most species was Compositae (5 species). The main medicinal parts were roots. The most frequently reported species were *Neopicrorhiza scrophulariiflora* (107 use reports), followed by *Gymnadenia orchidis* (75), *Artemisia calophylla* (72), *Fritillaria cirrhosa* (61), *Rhodiola himalensis* (48) and *Gastrodia elata* (46) (Fig. 4).

The FIC results for the 27 use categories ranged from 0.5714 to 0.9774, and the values of the FIC were the highest for respiratory system disorders (0.9774), followed by muscular–skeletal system disorders (0.9716), and the lowest for veterinary medicine (0.5714), followed by endocrine system disorders (0.8000) (Table 4).

**Table 4:** Evaluation of medicinal plants based on FIC

Categories of diseases	FIC
Poison	0.9412
Inflammation	–
Poisonings	–
Infections	0.9000
Digestive system disorders	0.8846
Respiratory system disorders	0.9774
Nutritional disorders	0.9626
Endocrine system disorders	0.8000
Muscular–skeletal system disorders	0.9716
Genitourinary system disorders	0.9444
Skin disorders	0.9362
Veterinary medicine	0.5714
Nervous system disorders	0.8333
Circulatory system disorders	0.9589
Eyes	–

### Wild economic plants

**Fig. 5:**
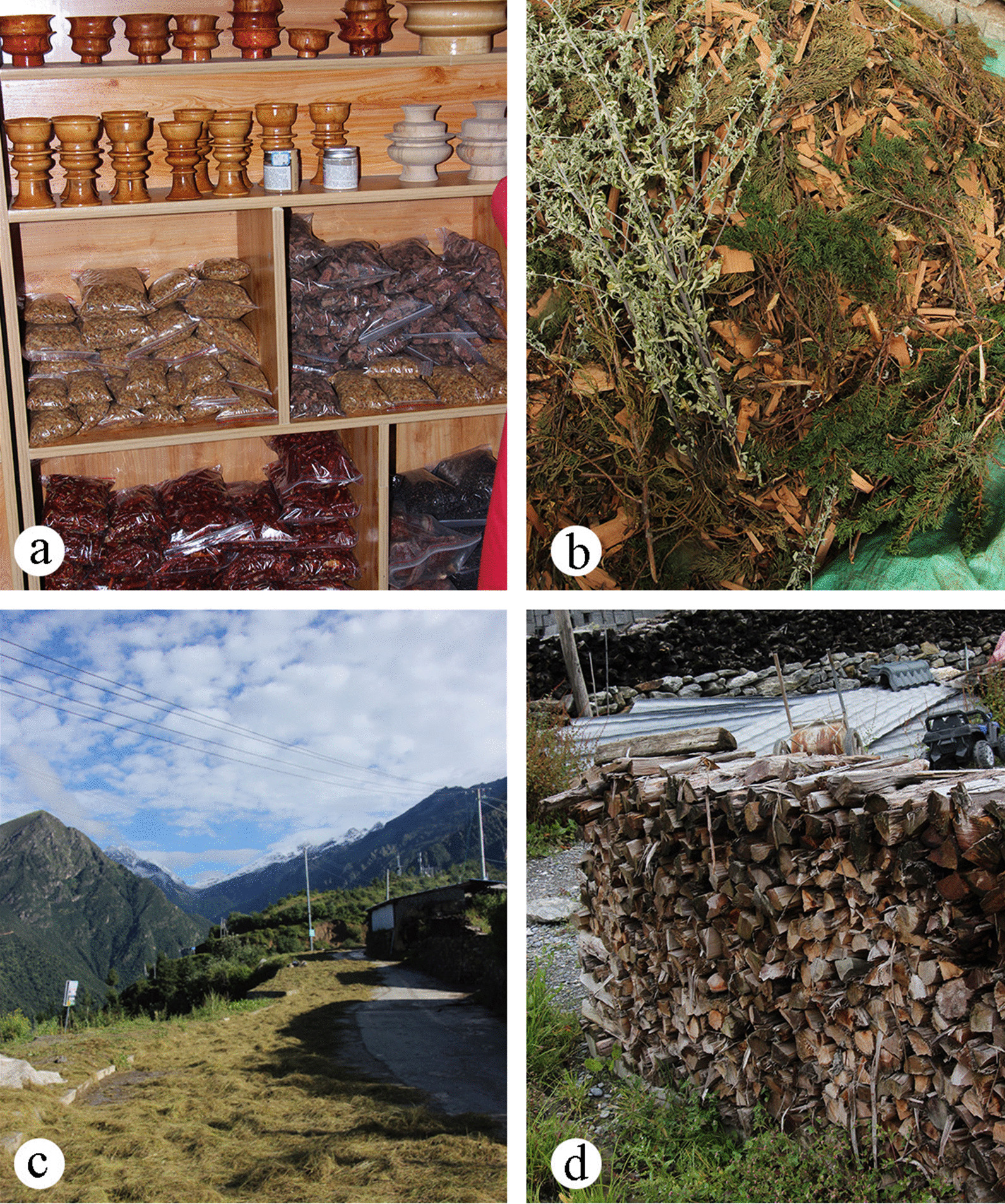
Other use categories. **a** some economic plants are sold in shops; **b** some plants used to “Sang”; **c** fodder plants; **d** fuelwoods

A total of 22 wild plants were used as economic plants (Table 3), and the most frequently reported species were *Fritillaria cirrhosa*. Wild economic plants are an important source of local income (Fig. 5a). A variety of economic plants are sold in local shops (Table 5). In addition to plants, there are *Cordyceps* sp. and wild *Ganoderma* sp. (Table 5).

**Table 5:** A price list from a shop in Gyirong

Botanical taxon	Local name(s)	Parts	Price (RMB/500 g)
*Zanthoxylum bungeanum* Maxim	ei1-ma1	Fruits	50/500 g
*Allium przewalskianum* Regel	zen1-bu1	Leaves	50/500 g
*Gastrodia elata* Blume	tian3-ma3	Roots	1200/500 g
*Polygonatum sibiricum* F.Delaroche	rang3-ma1-xia-jia1	Roots	190/500 g
*Gymnadenia orchidis* Lindl	wang1-bu1-la1-ba1	Roots	500/500 g
*Rhodiola himalensis* (D. Don) S.H. Fu	suo3-la1-ma3-bu4	Roots	150–750/500 g
*Fritillaria cirrhosa* D.Don	bai1-mu4	Bulbs	700–1000/500 g
*Rhododendron anthopogon* D. Don	po1-lu1	Flowers	50/500 g
*Pinus wallichiana* A.B.Jacks	tang3-xin1	Pollen	500/500 g
*Carum carvi* L	guo1-nie1	Seeds	50/500 g
*Taraxacum sikkimense* Hand.-Mazz	se4-ji4-mei3-duo3	Whole plant	80/500 g
*Solanum tuberosum* L	a3-lou3	Tuber	25/500 g
*Capsicum annuum* L	ku1-sa1	Fruits	50/500 g
*Ganoderma* sp.	po1-lu4-xia1-mo4	Fruiting body	600–1200/500 g
*Cordyceps sinensis* (BerK.)Sacc	ya1-za1-gong1-bu4	Fruiting body	30–50/piece
Wooden spatulas			10/piece
Wooden bowls			80–250/piece
Bamboo products			150–300/piece
Gourd ladle			25/piece
Tibetan incense powder			15 yuan/jar

### Other use categories

In total, 71 plants from other use categories, including ritual plants (24), fodders (20), fuelwoods (19), craft plants (10), tools (5) and dyes (4) (Table 3).

Tibetans burn some plants in their daily life to pray for happiness. A total of 22 wild plants were used for ritual use (Fig. 5b), and the most frequently reported species were *Rhododendron anthopogon*.

A total of 21 plant species were used as fodders (Fig. 5c), and the most frequently reported species were *Polygonum tortuosum*, followed by *Fargesia* sp. and *Poaceae* sp. Animal husbandry is one of the local important industries. In addition to grazing in pastures, local Tibetans also collect some plants and store them before the withered period arrives to supplement nutrition for livestock.

In addition, a total of 30 wild plants were used as fuelwood (Fig. 5d), tools, dyes and crafts. Among them, the most frequently reported is Rheum australe, which is used to dye clothes and wooden bowls. The locals collect its roots, dry them in the sun, boil them in water and put them in wooden bowls to dye them red. The making of wooden bowls is a symbolic handicraft culture of the Gyirong. They collect the stems of dead birch or cypress trees and process them into wooden bowl handicrafts, which is more well documented in our previous study [9].

### Comparison of wild useful plants between Tibetan ethnic groups in different areas

**Fig. 6:**
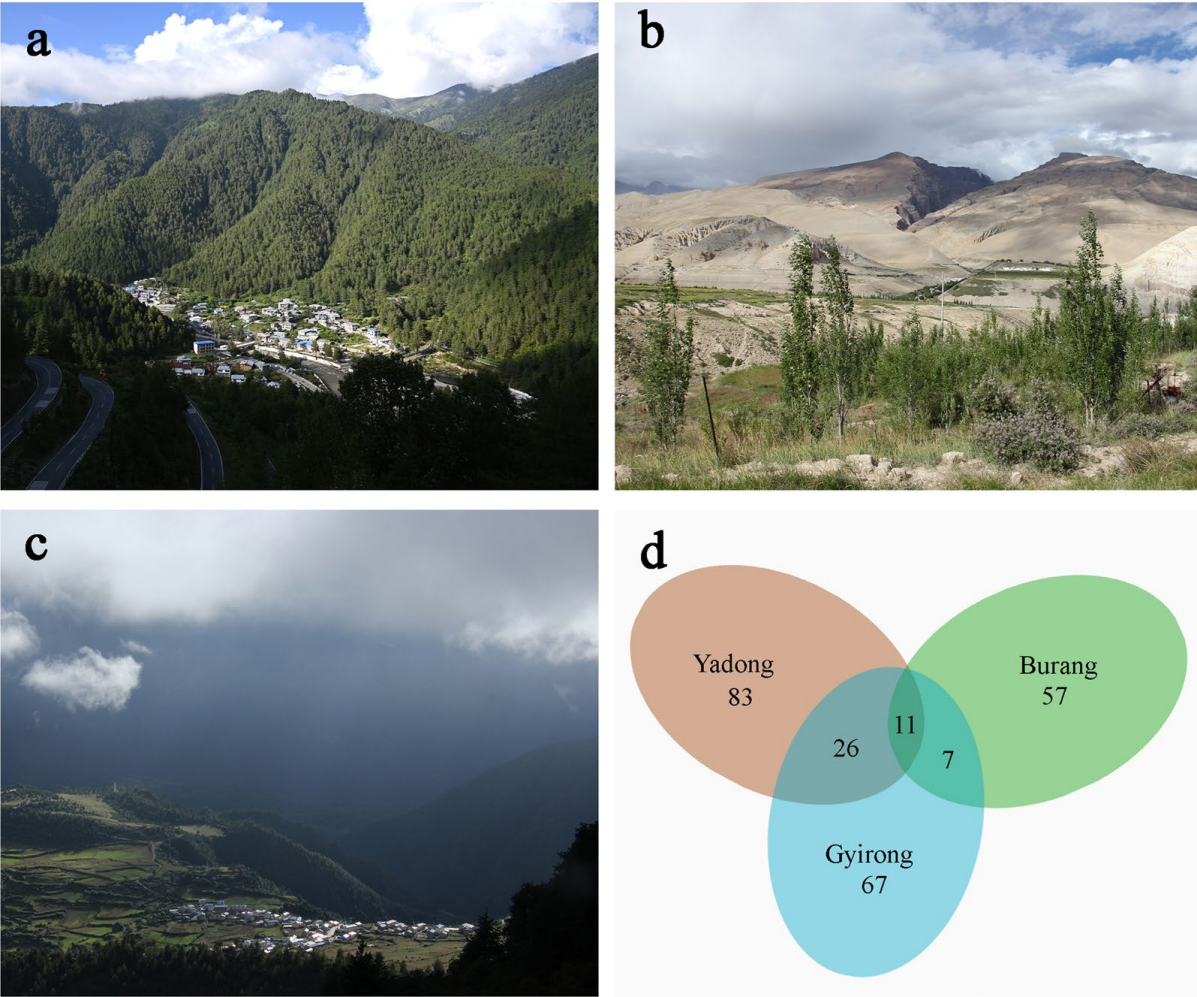
Comparison of wild useful plants between three Tibetan ethnic groups. **a** The natural landscape of Yadong; **b** the natural landscape of Buran; **c** the natural landscape of Gyirong; **d** Venn diagram of three communities

We mainly compared the differences in wild useful plant species among Gyirong (with a total of 110 species), Burang (with a total of 75 species) [27] and Yadong (with a total of 121 species) [22]. The results showed that 36 species of plants in the catalog of Gyirong and Yadong were the same, but only 17 species were the same in Gyirong and Burang. In addition, there were only 11 overlapping species between all the three regions. In general, the wild useful plant resources in Gyirong and Yadong are more abundant and similar than in Burang (Fig. 6d).

## Discussion

### Natural environment and culture influence indigenous plant knowledge

Firstly, these differences might be caused by the distribution of the plants. The research areas are not the same size, and the geographical and climatic environments and vegetation types are different [52]. Gyirong and Yadong include tropical to subtropical climate, and the main vegetation type is coniferous broad-leaved mixed forest (Fig. 6). However, Burang Town belongs to the temperate arid climate, and the main vegetation types are desert grassland (Fig. 6) [53]. Although there are differences in the utilization of plants in the three areas, there are still some plants that reflect the common preferences of them. For example, *Carum carvi*, an important wild vegetable and seasoning in Tibetan regions, ranked top 5 in CII value in all three regions. The use of Tibetan incense plants such as *Juniperus indica* and *Rhododendron anthopogon* also reflects the common cultural characteristics of the Tibetan people. In addition, *Neopicrorhiza scrophulariiflora* and *Saussurea tridactyla* also show the current situation of the spread of plant culture driven by the economy [22, 27].

To sum up, different natural environments may lead to different plant utilization. For example, there are obvious differences between Yadong, Gyirong and Burang, and each region has its own special plant knowledge. Previous studies have noted that geographical isolation could contribute to the preservation of diverse cultural traditions of local people in Himalayan regions [54] and could help preserve diverse traditional botanical knowledge. Our study also shows that the same cultural groups have common cultural preferences, for example, 11 plant species are shared across the three areas.

### Important wild useful plants

Based on the results of the CII quantitative analysis, we evaluated the top five wild plants that are important in the daily life of Tibetans in Gyirong Town.

*Allium prattii* C.H.Wright (CII = 1.071) is an important edible plant in Gyirong. Its young leaves and bulbs can be consumed as wild vegetables, and its fruits and flowers can be eaten as seasonings. A local woman said:

Ri guo (*A. prattii*) is a very delicious seasoning, you can use it for stewing potatoes or meat. There's a lot of it on the mountain that we pick it for consuming or selling.

This reflects two aspects of the plant, the first is that it is a delicacy that locals enjoy. In addition, it is easy to obtain. This plant is widely distributed in the Himalayas of China and northern India and Nepal [55]. It is also used as an edible plant in other areas. For example, it has the same usage as Gyirong in Yadong County, Tibet [22]. In Litang, Sichuan, China, the Tibetans also use the fresh bulb of the plant as a wild vegetable and spice [32]. In addition, it is also used to increase appetite and treat digestive system diseases, which was recorded in the Tibetan medical scripture “Jing Zhu Ben Cao” [56].

*Neopicrorhiza scrophulariiflora* (Pennell) D. Y. Hong (CII = 1.016) has important practical and economic value, which also drives the local people to collect it. Locals grind the dried root and drink it with boiling water to treat inflammation or fever. The plant is mainly distributed in the eastern Himalayas, at the junction of China and Nepal [55]. *Neopicrorhiza scrophulariiflora* was widely used by the locals to treat cold. This plant was first recorded in the “Si Bu Yi Dian and was mainly used to treat fever [23, 57]. According to the Chinese Pharmacopoeia, this plant can treat many diseases [42]. In the Yadong County of the Himalayas and Maithili region of eastern Nepal, it is used by locals to treat fever and headaches with high consensus [30].

*Gymnadenia orchidis* Lindl. (CII = 0.921), its root is an important tonic, the local people cook it with chicken, duck or milk, which can nourish the body. It is a traditional Tibetan medicinal plant for nourishing which was documented in “Jing Zhu Ben Cao” [56]. It was first recorded in the Tibetan medical work “Four Medical Canons” born in the eighth century AD [23]. The roots also were sold to increase income.

*Rhododendron anthopogon* D. Don(CII = 0.873)is an important ritual plant for “Sang” (People burn some plants in the morning to pray for a peaceful day) [58], the distribution range of *R. anthopogon* is almost all over the Himalayas, so is relatively easy to obtain [55]. An informant mentioned:

“We have to burn incense plants every morning, which smell good and can refresh us.”

When we ask what plants are best. He replied:

“Polu (*R. anthopogon*) is the best.”

The local Tibetans collect the old leaves of *R. anthopogon* and sun-dry them as materials for “Sang.” In addition to *R. anthopogon*, *Juniperus indica* and *Artemisia* sp. are important materials for “Sang.” *R. anthopogon* is also a beverage plant for local people to drink and sell. Its flowers are collected and sun-dried and then soaked in water to drink. It has a unique flavor, but drinking too much will cause headaches, which may be related to the toxic ingredients contained in it. According to the Chinese Pharmacopoeia, its flowers and leaves are used separately in Tibetan medicine, and its flowers can be taken as tea, which has a good curative effect on asthma and chronic bronchitis [59].

The bulbs of *Fritillaria cirrhosa* D. Don (CII = 0.857) were used by the locals to treat colds and coughs, and the main processing method is decoction. In addition, *F. cirrhosa* is also an important economic plant and a veterinary medicinal plant. It is recorded in the Chinese “Materia Medica and Tibetan Medicine Volume” that *F. cirrhosa* has the effect of resolving phlegm and relieving cough [23]. *Fritillaria cirrhosa*, which has high commercial value, has been excessively and indiscriminately harvested. As a result, its resources are declining sharply, and it is on the verge of extinction [60].

In particular, of these top five plant species, four were driven by economic value and one was driven by culture. This reflects to a certain extent that the main driving force for the spread of plant utilization knowledge is the economy.

### The state of traditional knowledge of wild useful plants in Gyirong

Tibetans of Gyirong have a wealth of knowledge. Most of the people who have acquired knowledge among the Tibetans of Gyirong are middle-aged, and these people have more voice and power in social life. Young people are reluctant to learn traditional plant knowledge [9]. Therefore, with the development of social economy and time, traditional knowledge is slowly disappearing or changing into other forms, such as knowledge about the economic plants. Protecting and documenting preexisting botanical knowledge is important and urgent.

Traditional wild plants’ knowledge of local Tibetans is also heavily influenced by traditional Tibetan medicine and tourism [9, 26]. The plant knowledge of the Tibetans in Gyirong is influenced by the traditional Tibetan medicine culture. In the cataloging of this study, 26 species were documented in traditional Tibetan medicine books [23]. In addition, locals sell many wild plants in the store, including various seasonings and medicines, and these products are mainly aimed at tourists. With the development of commerce, the excessive collection of plants has caused a certain degree of damage to the local ecological environment [61].

Local people not only use wild plants to meet their own needs but can also profit from wild plants. According to local government statistics on the basic situation of the township, the understory economy of wild plants has become an important source of economic income for locals. For example, *Fritillaria cirrhosa* and *Neopicrorhiza scrophula* are suffering from exhaustive collection. In addition, there are 11 other plant species under different levels of protection, but these plants are not protected because of commercialization [51].

Gyirong Tibetans have a rich traditional knowledge of wild plants, which has been influenced by the traditional Tibetan medicine culture. With the development of the social economy, their traditional knowledge of plants has also been affected by tourism culture, and the economy has gradually become the important driving force of wild plant collection.

### The relevance of this study for the development of the local community

Locally, the economy has become the main driver of the use of local plants. This phenomenon, if not restricted, may lead to the overharvesting of wild plants. Although local people obtain permits before collecting the fungus, there are no special management measures for collecting other wild plants. It is worth noting that the impact of the current tourism economy has made local traditional knowledge increasingly narrow. Previous studies have shown that biodiversity loss not only negatively affects the ecology and environment, but also culture, with profound implications for cultural resilience and biocultural diversity conservation efforts [62]. Therefore, if local communities want to achieve sustainable use of wild economic plants, we should not only pay attention to the protection of biodiversity, but also pay attention to the importance of traditional culture [63]. Local communities should carry out protection activities from the aspects of the restricted collection of economic plants and recording and publicizing traditional knowledge.

Although wild edible plants can provide additional nutritional supplements to local people, we should also pay attention to the possible harm of some plants when they are consumed. According to previous reports, *Pteridium aquilinum* var. *latiusculum* is a nutritious wild vegetable. If it is not soaked for enough time and cooked, the toxic carcinogens contained in the plant will not be removed [64, 65]. It contains Anthraquinones (AQs) in *Rheum australe*. An increasing number of studies have reported that AQs induce nephrotoxicity [66]. The young leaves of *Phytolacca acinosa* are used as wild vegetables, but the red roots of it are poisonous and inedible [55]. Therefore, the food safety of wild edible plants should also be an issue for community development.

## Conclusion

Gyirong has rich plant diversity and a long history and culture. This study is the first systematic cataloging and evaluation work using ethnobotanical survey and research methods in Gyirong. This study enriched the ethnobotanical study of the Himalayan region, and 111 wild plant species used in local Tibetan daily life were recorded. Multiple uses of these plants were analyzed, and the most culturally significant species of the local Tibetan people were identified by quantitative methods. Medicinal and edible plants play a significant role for the local Tibetan people in household-level food and health.

Based on the comparison study, the use of wild plants differed sharply among different areas, which might be attributed to the various geographical environments and vegetation types. In addition, people in different Tibetan communities retain similar plant use preferences. Botanical traditional knowledge of Tibetan in Gyirong is also heavily influenced by the traditional Tibetan medicine culture and tourism. In the future, we should pay more attention to the reasonable protection of cherished plants and promote the inheritance and development of traditional knowledge.

## Data Availability

Please contact the corresponding author for data requests.
